# Urinary Extracellular Vesicles as Biomarkers of Kidney Disease: From Diagnostics to Therapeutics

**DOI:** 10.3390/diagnostics10050311

**Published:** 2020-05-16

**Authors:** In O. Sun, Lilach O. Lerman

**Affiliations:** 1 Division of Nephrology and Hypertension, Mayo Clinic, Rochester, MN 55905, USA; inogood77@gmail.com; 2 Division of Nephrology, Department of Internal Medicine, Presbyterian Medical Center, Jeonju 54987, Korea

**Keywords:** urine, exosomes, biomarkers, kidney diseases

## Abstract

Cell-derived extracellular vesicles (EVs) can be isolated from various body fluids, including urine. Urinary EVs have gained important recognition as potential diagnostic biomarkers in renal disease since their cargo includes nucleic acids, proteins, and other cellular components, which likely mirror the physiological and possibly pathophysiological state of cells along the nephron. Accumulating evidence highlights the feasibility of using EVs as biomarkers for diagnostic, prognostic, and therapeutic purposes in several forms of renal disease, such as acute kidney injury, glomerulonephritis, and renal transplantation. Additionally, exogenous delivery of EVs released in vitro by cells in culture may have salutary benefits for renal diseases. In this review, we introduce recent studies that attempt to identify urinary EVs as candidate biomarkers for human kidney diseases and consider their potential implication as a therapeutic option in key kidney diseases.

## 1. Introduction

Extracellular vesicles (EVs) are membrane-bound nanosized structures generated and released into the extracellular fluid by cells under stressful conditions. They serve the role of intercellular communication and transfer their cargo to alter the phenotype of the recipient cells [1]. EVs contain various molecules, including proteins, lipids, DNA, mRNA, and microRNA (miRNA), and are characterized by the common proteins that they carry, including tumor susceptibility gene-101, tetraspanins (CD9, CD63), heat shock proteins, annexins, and apoptosis-linked gene-2-interacting protein-X. Additionally, EVs can also display surface markers from their cell of origin, such as aquaporin-2 in the collecting duct, sodium/hydrogen exchanger-1 in the proximal tubule, and podocalyxin in podocytes. The main types of EVs are exosomes, microparticles, and apoptotic bodies, which are distinguishable by their cellular origin, size, and cargo [2,3].

EVs have been detected in blood and urine, as well as in other body fluids. Urine is a highly useful specimen for biomarker discovery that is used to diagnose and monitor kidney diseases because it can be collected repeatedly using non-invasive techniques. Proteomic analysis has shown that the majority of urinary EV cargo represents glomerular, tubular, prostate, and bladder cells, whereas circulating EVs presumably cannot cross the filtration barrier, at least under physiological conditions, supporting the notion that urinary EVs derive primarily from cells in the genitourinary tract facing the urinary space [4]. Therefore, analysis of urinary EVs may serve as a logical and novel diagnostic approach in kidney disease since changes in the number or characteristics of released EVs may be linked to the development of disease or response to therapy.

## 2. Urinary EV Isolation

The isolation of urinary EVs needs to take into account several practical considerations. For studies using urine EVs, it is important to maintain optimal storage conditions of urine samples to prevent proteolysis. Storage at −80 °C, rather than 4 °C or −20 °C, is preferable to prevent degradation, although the use of freshly processed urine is most optimal. Urine can be collected as a spot or timed sample, and the process of EV isolation begins with a low speed and/or a low centrifugal force (3000× *g*) centrifugation step for a short time (< 10 min) and at a low temperature (4 °C) [5]. Then, urine is carried forward through to the urinary EV isolation step. Several methods for isolating EVs have been described and frequently used. Traditionally, EVs are isolated and purified using differential centrifugation and ultracentrifugation. However, ultracentrifugation is not only labor-intensive and time-consuming but also requires expensive laboratory equipment, making it impractical for high throughput clinical applications. Ultrafiltration represents a faster and simpler method of isolating urinary EVs and usually involves the use of a polyethersulfone nanomembrane filter [5]. However, this method is inefficient in patients with nephrotic syndrome because of protein adherence to the nanomembrane and high protein retention. Precipitation followed by centrifugation has also been explored for rapid exosome isolation. Several commercial precipitation reagents have been introduced over the last few years. Kits such as ExoQuick-TC^TM^ and Total EX isolation reagent from Invitrogen^TM^ are based on aggregating agents followed by low-speed centrifugation [6]. Isolation of urinary EVs using a commercial kit is quicker than other methods because it does not require ultracentrifugation, yet may be more costly. The amounts of EVs collected may also vary between isolation methods and has been reported to be around (2–4) × 10^8^ particles/mL of urine [7].

## 3. Urinary EVs as Diagnostic Biomarkers for Kidney Diseases

### 3.1. Acute Kidney Injury

Most cell types in the kidney produce and secrete EVs. Proteomic analysis of urinary EVs has confirmed that proteins within them may arise from all nephron segments, including podocytes, proximal tubules, the thick ascending limb of Henle’s Loop, the distal tubule, and the collecting duct. Therefore, exosomal proteins may be leveraged as biomarkers for location-specific diseases [4]. The exosomal content may vary in response to various pathophysiological conditions such that exosomal proteins may be biomarkers for specific diseases. For example, the aquaporin-1 (AQP1) protein level in urinary exosomes has been identified as a potential biomarker for acute kidney injury (AKI). During renal ischemia/reperfusion in rats, urinary exosomal AQP1 decreased significantly [8]. Similarly, the Na^+^/H^+^ exchange type-3 and fetuin-A levels in urinary exosomes have been identified as potential biomarkers for AKI. Increased levels of Na^+^/H^+^ exchange type-3 in urinary exosomes were observed in acute tubular necrosis but not in prerenal azotemia or other causes of AKI, which may help differentiate those causes of AKI [9]. In a rat model of cisplantin-induced AKI, a marked increase in urine exosome fetuin-A was detected not only before an elevation in serum creatinine levels but also before any evidence of morphological injury. Zhou et al. suggested the diagnostic utility of this biomarker in a limited number of intensive care unit patients with AKI [10]. Additionally, urinary levels of the exosomal transcription factor ATF3 increased in AKI patients before the elevation of serum creatinine, indicating that ATF3 may be useful as a biomarker for the early diagnosis of AKI [11]. Subsequently, urine ATF3 was also shown to be a biomarker for sepsis-induced AKI [12]. Therefore, elevated urinary levels of a specific exosome might provide an early index of etiology-specific AKI.

### 3.2. Glomerular Disease

Urinary biomarkers may mitigate the need for an invasive kidney biopsy in glomerular disease. Podocytes, which are specialized epithelial cells forming the glomerular filtration barrier with the glomerular basement membrane, are the main cell-types affected in glomerular disease. Thus, podocyte-derived EVs may be a promising index of glomerular injury. In focal segmental glomerulosclerosis (FSGS), a disease with significant podocyte damage, Wilm’s tumor-1 (WT-1), a transcriptional factor required for normal kidney development, was examined in urinary exosomes as a marker for podocyte damage. Increased expression of WT-1 in urinary exosomes preceded urinary albumin excretion by one week in a mouse model of collapsing glomerulopathy [13]. Additionally, in human subjects with FSGS, exosomal WT-1 levels were significantly higher in children with active nephrotic syndrome caused by FSGS compared with healthy controls or patients with steroid-sensitive nephrotic syndrome (SSNS). Furthermore, a significant fall in urinary exosomal WT-1 was also shown in patients in remission of SSNS following steroid treatment [13]. WT-1 expression in urinary EVs was also found to be elevated in patients with diabetes, in which podocyte injury is an early feature. Kalani et al. identified WT-1 in urinary exosomes of diabetic patients, which correlated with declining renal function, highlighting exosomal WT-1 as a biomarker of podocyte injury in diabetic nephropathy [14]. Notably, we recently identified elevated numbers of podocyte-derived exosomes in obese compared with lean human subjects and pigs [15], which might represent early markers of kidney disease.

Urinary EVs may also help to differentiate early IgA nephropathy from thin basement membrane nephropathy. Moon et al. used proteomic analysis to compare levels of exosomal proteins in these two diseases and found that levels of aminopeptidase-N and vasorin precursor were higher in the thin basemen-membrane nephropathy group, whereas α-1-antitrypsin and ceruloplasmin levels were elevated in IgA patients [16]. Moreover, urinary exosomal chemokine ligand-2 (CCL2) levels are upregulated in IgA nephropathy patients compared with healthy controls and correlate with renal function and histologic injury [17]. Hence, different forms of glomerular disease might show specific profiles of urinary EV protein expression.

### 3.3. Tubular Disease

Urinary EVs often contain solute transporters, such as Na-Cl cotransporter (NCC), which was found to be absent in patients with Gitelman’s syndrome or Bartter syndrome type-1 [18], a genetic disorder caused by a mutation of the gene encoding for this cotransporter-2 [19]. Yet, while urinary EV phenotyping might be useful in the diagnosis of Gitelman’s and Bartter syndromes [20], additional studies are needed to address whether these EVs can be used as a preliminary screening tool in patients suspected of having these genetic disorders.

Polycystic kidney disease (PKD) is the most common inherited kidney disease and is caused by a mutation in genes encoding for proteins involved in the function of primary cilia, including polycystin (PC)-1, PC-2, and fibrocystin. EVs may be important elements in cilia biology and biomarkers for PKD, as cystin and ADP ribosylation-factor-like-6 are abnormally expressed in urinary EVs of patients with PKD [21]. Recently, the presence of transmembrane protein-2 (TMEM2) in urinary exosomes was shown to be more than twofold higher in patients with PKD1 mutations compared with controls. The PC-to-TMEM ratio correlated inversely with kidney volume, suggesting that urine exosomal PC-1/TMEM2 or PC-2/TMEM2 may provide a novel technique to assess disease progression in patients with PKD. Recently, levels of plakins and complement in urinary EVs have also been implicated as disease-associated proteins in autosomal dominant PKD [22].

### 3.4. Aging

Aging is associated with a high prevalence of chronic degenerative diseases that impose an increasing burden of morbidities in a growing demographic of elderly people. Circulating plasma levels of EVs decrease with age, possibly due to increased internalization of vesicles into B-cells [23]. Aging also involves activation of both autonomous and non-autonomous cell mechanisms, a dominant among which is senescence. Cellular senescence is a form of cell-cycle arrest that changes cellular characteristics toward a pro-inflammatory phenotype. Regarding urinary EVs, six proteins (TSN1, PODXL, IDHC, PPAP, ACBP, and ANXA5) were found to express differently between subjects that were 25–50 and 50–70 years old [24]. Furthermore, the numbers of urinary exosomes, juxtaglomerular cells, and podocyte marker-positive EVs in human subjects decrease with increasing age, and several populations of EVs derived from glomerular and other nephron cells correlate with the development of nephron hypertrophy or nephrosclerosis [25]. Recently, we also found that mitochondrial dysfunction might be associated with cellular senescence in the early stage of atherosclerotic renal artery stenosis [26]. In short, aging impacts the renal cellular composition and its urinary representation.

### 3.5. Renovascular Disease

Renovascular disease (RVD) is the main cause of secondary hypertension, and prolonged renal ischemia may result in irreversible kidney damage [27]. Kwon et al. reported differential expression of miRNA in urinary EVs obtained from hypertensive patients [28]. The level of miRNA-21, -93, and -200b were lower in urinary EVs of patients with essential hypertension than in RVD, suggesting them as useful markers for discriminating different forms of renal damage in hypertension. Furthermore, urinary podocyte-derived EVs were also elevated in RVD patients and might be useful for monitoring glomerular damage in these patients [29]. Subsequently, we identified elevated urinary levels of exosomes derived from p16^+^ tubular cells in hypertensive patients, suggesting increased tubular cellular senescence [30], and from peritubular capillaries, reflecting renal microcirculation injury [31].

### 3.6. Renal Transplantation

Renal transplantation is the best option for end-stage renal disease. The use of urinary EVs as biomarkers for kidney injury after renal transplantation was reported by Alvarez et al., who demonstrated the presence of neutrophil gelatinase-associated lipocalin (NGAL), a member of the lipocalin family that is largely expressed in tubular cells in response to inflammation, in cellular fraction and urinary exosomes from transplanted patients [32]. Despite no change in urinary free NGAL levels, its levels in urinary exosomes distinguished grafts harvested from deceased compared to living donors, and increased in patients with delayed graft function, suggesting that urinary exosomal NGAL could be a biomarker of injury or delayed graft function after kidney transplantation.

Decreased urinary CD133^+^ EV levels are also associated with delayed graft function and vascular damage [33]. These CD133^+^ EVs were detectable at high levels in urine from healthy donors but not in patients with end-stage renal disease, possibly indicating that these vesicles are only released by functioning renal tissue. In transplant patients, urinary CD133^+^ EVs are present at low levels on the first day after transplantation and increase thereafter at day 7. Contrarily, in patients with severe pre-transplant vascular damage of the graft, CD133^+^ EVs do not increase on day 7. Thus, CD133^+^ EVs may reflect restored tubular function and the regenerative potential of the nephron after renal transplantation.

Intra-graft infiltration of T-cells is one of the hallmarks for the diagnosis of acute cellular rejection after kidney transplantation. A recent study showed that CD3-positive urinary exosomes in patients with acute cellular rejection could reflect T-cell infiltration in the allografts [34]. In the case of antibody-mediated rejection, mRNA transcript profiles, such as gp130, SH2D1B, TNFα, and CCL4, were increased in plasma exosomes of the patients [35].

## 4. EVs as a Therapeutic Approach in Kidney Disease

Mesenchymal stem cells (MSCs) are multipotent cells with robust self-renewal, regenerative, proliferative, and multi-lineage differentiation potential. Harvesting and delivery of MSC-derived EVs emerged as a novel non-cellular alternative to cell-based therapy. A potential therapeutic role of EVs in AKI has been examined extensively in animal models. Several studies have shown that MSC-derived EVs confer renoprotective effects in kidney injury models. The first evidence of the beneficial effect of EVs shed by bone-marrow MSC was reported by Bruno et al. in 2009, in a model of AKI induced by glycerol injection in immune-deficient mice [36]. They reported that MSC-derived EVs improved tubular injury and renal function by inducing tubular cell proliferation and that this may occur via horizontal transfer of mRNAs by the EVs. Recently, our group tested intrarenal delivery of autologous MSC-derived EVs in a porcine model of metabolic syndrome and renal artery stenosis. We found that intrarenal injection of MSC-derived EVs in these pigs ameliorated renal inflammation, increased the number of reparative macrophages, and upregulated expression of IL-10, suggesting that the protective effects of EVs on the stenotic kidney are due to anti-inflammatory properties [37]. Furthermore, intra-renal delivery of MSC-derived EVs bearing pro-angiogenic properties restored renal microcirculation, hemodynamics, and function in an experimental animal model [38]. Interestingly, we subsequently found that intrarenal injection of MSC-derived EVs was helpful to mitigate the myocardial injury in experimental RVD likely via improvement of kidney function [39]. Furthermore, a recent study revealed that the delivery of exosomes secreted by urine-derived stem cells was useful in alleviating stress urinary incontinence and kidney complications from type-I diabetes in rats [40]. Additional studies are needed to determine whether the delivery of other types of exosomes isolated from the urine can serve as therapeutic tools in human subjects as well [41].

## 5. Urinary EVs as Monitors of Drug Therapy

Recently, circulating EVs, especially tumor-derived exosomes, have been evoking interest as surrogates of response to anti-cancer therapy, as well as diagnostic biomarkers. For example, circulating microvesicles allow for real-time monitoring of cancer’s response to chemotherapy [42,43]. However, few studies have assessed the role of urinary EVs in monitoring the effects of drug therapy. In essential hypertensive patients, hydrochlorothiazide-induced reduction of blood pressure is correlated with NCC abundance in urinary exosomes [44]. The increase in NCC abundance in urinary exosomes was detected in responders but not in non-responders, implying that characteristics of urinary EVs may help direct therapy. More studies are needed to investigate the role of urinary EVs as a monitoring tool in drug therapy.

## 6. Urinary EVs as Vehicles for Drug Delivery

Recently, EVs have gained prominence as drug delivery vehicles thanks to their ability to deliver proteins, lipids, and nucleic acids to recipient cells [45]. Exosomes provide important advantages compared to other nanoparticle drug delivery systems, such as liposomes and polymeric nanoparticles [46]. Different cell sources for EVs have been investigated because the parental cell has been shown to influence their biological activity and subsequent therapeutic effect [47]. These include model cell lines, such as various tumor cell lines, dendritic cells from bone marrow, and mesenchymal stem/stromal cells.

The process of loading EVs with specific cargos can be attained by manipulating their parental cells (endogenous loading), or the isolated EVs (exogenous loading) themselves when endogenous loading is infeasible. Indeed, these versatile approaches may expand the applications of EVs of a wider variety of sources. Zhuang et al. reported that exosomes effectively delivered curcumin to the brain to treat neuroinflammation-related diseases, without adverse effects [48]. Endogenous loading techniques leverage the biological cellular apparatus to sort and pack molecules into EVs during their biogenesis. For example, incubating parental cells with chemotherapeutic paclitaxel and doxorubicin led to their incorporation [49,50] such that EV-encapsulated drugs could be isolated from the culture-conditioned media. Furthermore, to boost their delivery and biodistribution, EVs can be engineered to recognize specific cell surface receptors [51]. Thus, EVs can be loaded with therapeutic substances for delivery to target cells, exciting developments that may be used in future clinical trials. Whether these would apply to EVs isolated from urine remains to be tested.

## 7. Summary

**Figure 1:**
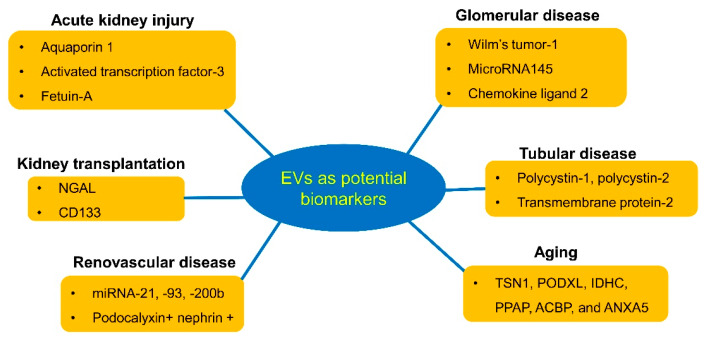
Potential biomarkers of urinary extracellular vesicles (EVs) in kidney diseases. Urinary EVs may augment the ability to diagnose and monitor renal conditions. Their most useful application may be predicting the clinical outcome of patients with kidney disease and guiding treatment decisions.

Investigation of various types of EVs is currently among the most stimulating areas in cell biology. In the field of kidney biology and disease, studies have particularly centered on the potential of EVs as biomarkers, focusing primarily on EVs detected in urine (Figure 1).

Such urinary EVs are appropriate and useful for diagnosis given the ease of collection and ability to serve as a “liquid biopsy” to provide information on the pathophysiological state of kidneys. The availability of a reliable and quick urinary assay of EV biomarkers might support a non-invasive diagnosis and facilitate adequate individualized management approaches. Additionally, further studies showing the relationship between the number of EVs and renal diseases are needed. The use of EVs as indices of the response to therapy is underutilized and needs further validation. Rigorous studies to detect and portray EVs will also extend our understanding of their diverse roles in health and disease, and provide new insight into the origins, diagnosis, and treatment options for renal disease.

## Abbreviations

AKI	acute kidney injury
AQP1	aquaporin-1
CCL2	chemokine ligand-2
EV	extracellular vesicle
FSGS	focal segmental glomerulosclerosis
MiRNA	microRNA
MSC	mesenchymal stem cell
NCC	Na-Cl cotransporter
NGAL	neutrophil gelatinase-associated lipocalin
PC	polycystin
PKD	polycystic kidney disease
RVD	renovascular disease
SSNS	steroid-sensitive nephrotic syndrome
TMEM2	transmembrane protein-2
WT-1	Wilm’s tumor-1
